# Wii-Fit for Improving Gait and Balance in an Assisted Living Facility: A Pilot Study

**DOI:** 10.1155/2012/597573

**Published:** 2012-06-13

**Authors:** Kalpana P. Padala, Prasad R. Padala, Timothy R. Malloy, Jenenne A. Geske, Patricia M. Dubbert, Richard A. Dennis, Kimberly K. Garner, Melinda M. Bopp, William J. Burke, Dennis H. Sullivan

**Affiliations:** ^1^Reynolds Department of Geriatrics, University of Arkansas for Medical Sciences, Little Rock, AR, USA; ^2^Geriatric Research Education and Clinical Center (GRECC), Central Arkansas Veterans Health Administration System, 2200 Fort Roots Drive (3J/NLR), North Little Rock, AR 72114, USA; ^3^Department of Psychiatry, University of Arkansas for Medical Sciences, Little Rock, AR, USA; ^4^Department of Family Medicine, University of Nebraska Medical Center, Omaha, NE, USA; ^5^Department of Psychiatry, University of Nebraska Medical Center, Omaha, NE, USA

## Abstract

*Objectives*. To determine the effects on balance and gait of a Wii-Fit program compared to a walking program in subjects with mild Alzheimer's dementia (AD). *Methods*. A prospective randomized (1 : 1) pilot study with two intervention arms was conducted in an assisted living facility with twenty-two mild AD subjects. In both groups the intervention occurred under supervision for 30 minutes daily, five times a week for eight weeks. Repeated measures ANOVA and paired *t*-tests were used to analyze changes. *Results*. Both groups showed improvement in Berg Balance Scale (BBS), Tinetti Test (TT) and Timed Up and Go (TUG) over 8 weeks. However, there was no statistically significant difference between the groups over time. Intragroup analysis in the Wii-Fit group showed significant improvement on BBS (*P* = 0.003), and TT (*P* = 0.013). The walking group showed a trend towards improvement on BBS (*P* = 0.06) and TUG (*P* = 0.07) and significant improvement in TT (*P* = 0.06). *Conclusion*. This pilot study demonstrates the safety and efficacy of Wii-Fit in an assisted living facility in subjects with mild AD. Use of Wii-Fit resulted in significant improvements in balance and gait comparable to those in the robust monitored walking program. These results need to be confirmed in a larger, methodologically sound study.

## 1. Introduction

Falls are common in Alzheimer's dementia (AD) and lead to significant morbidity and mortality [1]. Compared to cognitively intact older adults, patients with AD have a threefold increase in falls causing fractures, hospitalization, and increased rate of institutionalization [2]. Poor balance and gait abnormalities seen in AD such as shortened step length, increased stride length variability, and decreased gait speed are risk factors for falls [3, 4]. 

Exercise interventions improve gait and balance in the elderly [5, 6]. Even low-intensity exercises are useful in improving balance and gait in deconditioned elders [7]. However, it is difficult to engage patients with AD in long-term exercise programs. Barriers to exercise programs include but are not limited to lack of motivation, limited access to exercise facilities, and high cost of exercise programs [8]. Exercise programs are most effective if they are easy to access and perform. Walking, for example, is a popular activity and the most commonly recommended home-based exercise for the elderly [9]. Likewise use of readily available technologies might enhance exercise adherence because of their easy access, user friendly interface, and ability to provide high level of engagement at an affordable price. While off-the-shelf gaming technologies lack specificity, they have the advantage of mass accessibility, broad affordability, and the potential for in-home use. Wii-Fit, a Nintendo gaming console, is one such ready-to-use technology that provides self-directed activity through a TV [10]. The components include yoga, strength training, aerobics, and balance games. Virtual trainers talk the user through the activity while tracking their progress and providing visual and auditory feedback.

The primary goal of our study was to compare the effect of the Wii-Fit program to a walking program on balance and gait in subjects with mild Alzheimer's dementia living in an assisted living facility. To our knowledge there are no such previous studies.

## 2. Methods

### 2.1. Study Design, Setting, and Participants

A prospective randomized study with two intervention groups was conducted in an assisted living facility after obtaining approval from Institutional Review Board (IRB). All subjects signed a written assent while their legal surrogates signed a written informed consent. Subjects aged 60 years and older residing in an assisted living facility with a medical chart documenting a history of mild AD along with a Mini Mental State Examination score ≥18 (upper limit 29) were included in the study. Individuals were excluded for the following medical problems: myocardial infarction, transient ischemic attack or stroke in the previous 6 months, serious mental illness which impacted memory, active cancer diagnosis with the exception of skin cancer, poor prognosis for survival (e.g., severe congestive heart failure), severe sensory (visual or auditory) or musculoskeletal impairments, or a required use of a wheel-chair for ambulation. However, subjects using canes or walkers were included in the study. The walkers wrap around the Wii balance board so they used the walker while exercising.

### 2.2. Study Intervention

 Subjects were randomized using a random number generator to either the Wii-Fit intervention group or the walking group. The goal was for subjects in both groups to participate in the activity for 30 minutes daily, five times a week, for 8 weeks. The Nintendo Wii-Fit console was connected to a mobile television unit which was transported daily to the exercise area of the assisted living facility. Research personnel set up and navigated the Wii-Fit console and software, and the subjects performed the exercises. All subjects walked down from their rooms to the exercise area to do Wii-Fit and walked back to their rooms after the exercise. This walk from and to the room was included as a warm-up and cool-down exercise. Similarly, the walking group walked to the start point to begin their walking exercise and the walk from and to their room was included as warm-up and cool-down exercise.

 Based on the physical therapists recommendation, we encouraged patients to do exercises from each group of the Wii-Fit program that included strength training, yoga, and balance games. The Yoga exercises included the half-moon, warrior pose, chair, and sun salutation. Strength training included single leg extensions, lunges, and torso twists. Balance exercises included soccer heading, ski slalom, ski jump, table tilt, balance bubble, and penguin slide. Since one third of the participants used some kind of assistive device, the aerobic exercise such as the running and step activity of the Wii-Fit were not used per recommendation of the physical therapist. The Wii-Fit intervention was an individual exercise program wherein subjects exercised one on one with help from research personnel. In the Wii-Fit group, all subjects spent 10 minutes on yoga, 10 minutes on strength training, and 10 minutes on the balance games. This was standardized across all the participants. They were allowed to rest only if they became unduly fatigued. The walking arm walked at their own pace as a group of 3 or 4 subjects at any given time with research personnel. Again subjects were allowed to rest if they became unduely fatigued. All walking was done indoors. Subjects in both groups were monitored closely during participation to ensure their safety.

### 2.3. Outcome Measures

Demographic and anthropometric data were recorded. The primary outcome measure was the Berg Balance Scale (BBS). Tinetti Test (TT) and the Timed Up and Go (TUG) were also measured. The BBS [11] is a valid and reliable measure used to assess balance impairments in elderly, either statically or while performing various functional movements in a clinical setting. The BBS consists of 14 subsets of tasks with each task scored on a five-point scale (0–4) according to the quality or the time of the performance. A score of “0” indicates the lowest level of function while “4” indicates the highest level of function. The maximum score is 56, and a score of less than 45 is predictive of falls. The TT [12] is scored on the patient's ability to perform specific tasks. Scoring is done on a three-point ordinal scale with a range of 0 to 2. The individual scores are then combined to form three measures; an overall gait assessment score, an overall balance assessment score, and a gait and balance score. The TUG [13] test is widely employed in the examination of elders as a basic test for functional mobility. The TUG score measures speed and represents the time taken to rise from the chair, walk 3 meters, turn, walk back, and sit down. The BBS, TT, and TUG were performed by the same physical therapist.

The secondary outcome measures measured were functional ability, quality of life, and cognition. Functional ability was measured by activities of daily living (ADL) [14], and instrumental activities of daily living (IADL) [15]. ADL assesses a subject's independence in performing basic tasks of daily living such as bathing, eating, and toileting, while IADL assesses the subject's independence in performing hierarchical activities, such as preparing meals, handling finances, and using telephone. The higher the score of ADL and IADL, the better the functional state of the subject. Quality of life was measured by the Quality of Life-AD (QOL-AD) [16]. The QOL-AD is a 13-item scale which assesses patient's physical condition, mood, interpersonal relationships, ability to participate in meaningful activities, financial situation, and overall assessment of self and quality of life as a whole. Cognition was measured by the Mini Mental State Examination (MMSE) [17].

### 2.4. Statistical Analysis

The data were analyzed using SPSS (version 17.0 for Windows, SPSS Inc., Chicago, IL, USA). Each variable was analyzed and was found to have a normal distribution. Demographics and comorbidities were presented using descriptive statistics. Independent sample *t*-tests were used to test for intergroup differences at baseline. In the primary analyses, repeated measures analyses of variance (RM-ANOVA) were used to determine if there was a significant group by time interaction for any outcome when comparing the Wii-Fit and the walking groups over the three time points of the study, baseline and weeks 4 and 8. An alpha value of 0.5 was used to indicate significance. Post-hoc analyses using paired *t*-tests were performed on each group to determine within subject changes between the baseline and postintervention values. Data were analyzed with intent-to-treat analyses. For subjects who did not complete the study, the last data were carried forward to week 8.

## 3. Results

**Figure 1:**
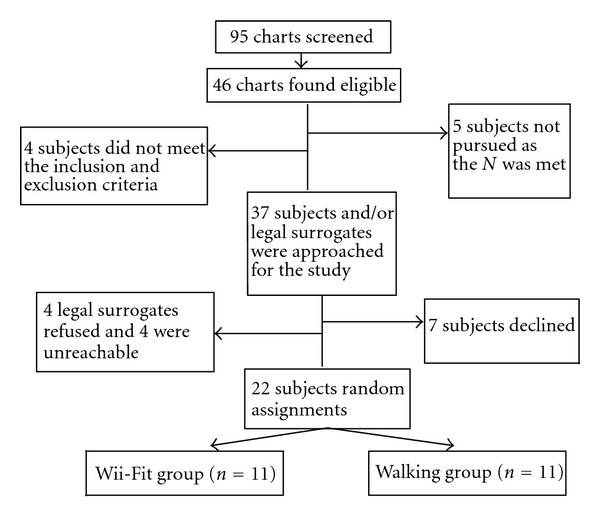
Subject recruitment and participation.

After completing the baseline assessments, subjects (*N* = 22) were randomly assigned to either the Wii-Fit Intervention or the walking group (*N* = 11 each). Study recruitment and participation are illustrated in Figure 1. One subject in each group completed just 4 weeks of the study. The subject in the Wii-Fit group voluntarily withdrew while the subject in the walking group was discharged from the facility to his home. There were no adverse events related to the study. However, unrelated adverse events occurred for several subjects. Two subjects' sustained falls (one from each arm), but these events did not occur during the intervention and did not require an emergency room visit or hospitalization. They continued to exercise without any difficulty. One subject was hospitalized for small bowel obstruction which resulted in the subject being much weaker and requiring the use of a walker upon return to the study. Two subjects (one from each arm) developed viral gastroenteritis and were quarantined for a total of four days, two days of which were their exercise participation days during which time they could not participate in the exercise activities.

The characteristics of the study subjects are summarized in Table 1. There were no statistically significant differences between these groups for any of the variables listed. There were also no significant differences between groups for the number of comorbidities present, medications prescribed, or for the need of assistive walking devices. About a third of the subjects used an assistive walking device. The total time of participation in the study interventions (used as an indicator of compliance) was not significantly different between the Wii-Fit and walking groups (Table 1). The Wii-Fit and walking were well accepted by the participants. They reported enjoying Wii-Fit exercises. The most common reasons for not participating in exercise activities included other scheduled activities such as field trips, bus rides, crafts and decorations, family visits, and doctor's appointments.

**Figure 2:**
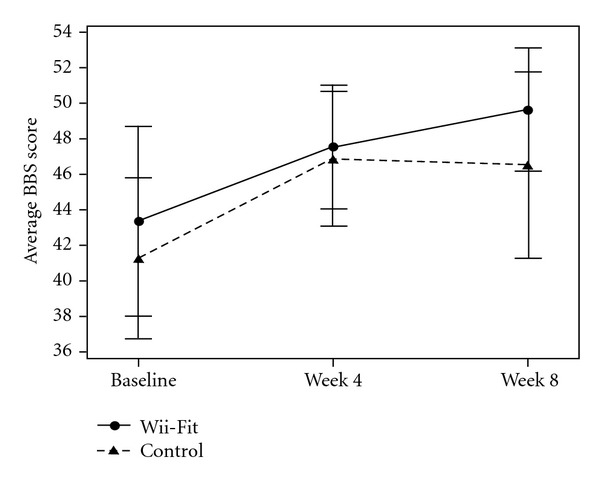
Comparison of mean change in BBS* between the Wii-Fit and walking groups. *BBS: Berg Balance Scale; Data points represent means and error bars represent ±2 SEM.

 The mean BBS score in both groups was less than 45 indicating that the subjects were at high risk for falls. The mean BBS score improved to more than 45 in both groups with intervention. BBS improved significantly over time for both groups (*P* = 0.0001). However, there were no significant group-by-time interactions on the measures of BBS (*P* = 0.56). There was also no significant group-by-time interactions on the measures of TT (*P* = 0.97) and TUG (*P* = 0.52) (Table 2). Intragroup analysis of the Wii-Fit group showed significant improvement on measures of BBS (average improvement 6.27 ± 5.27, *P* = 0.003), and TT (average improvement 1.82 ± 2.04, *P* = 0.013) (Table 2). Scores on TUG improved with Wii-Fit but did not reach statistical significance (average improvement −0.82 ± 2.56, *P* = 0.31) (Table 2). Intragroup analysis of the walking group showed a trend towards improvement on the BBS (average improvement 5.27 ± 7.36, *P* = 0.06) and TUG (average improvement −2.1 ± 3.45, *P* = 0.07) and significant improvement on TT (average improvement 2.0 ± 1.89, *P* = 0.006) (Table 2). The mean BBS improved nonsignificantly over the first 4 weeks in both groups, however the walking group plateaued from week 4 to 8 while the Wii-Fit group continued to improve (Figure 2). There was no significant group-by-time interaction on measures of functional state, ADLs (*P* = 0.11), IADLs (*P* = 0.11), quality of life (QOL-AD) (*P* = 0.61), or cognition (MMSE) (*P* = 0.7). However, intragroup analysis of walking group showed improvement on the measures of quality of life (*P* = 0.03).

## 4. Discussion

This study shows that the use of Wii-Fit is feasible in an assisted living environment in elderly patients with mild dementia. Those in the Wii-Fit group readily accepted and enjoyed participating in the exercise program. The mean baseline BBS score in both groups was less than 45 indicating that all the subjects were at high risk for falls. There were no study-related adverse events reported during the intervention. Thus the use of Wii-Fit was also safe and feasible in subjects at high risk for falls. The mean BBS score improved to more than 45 in both groups with intervention. These results demonstrated that the Wii-Fit program was as effective as the robust monitored walking program in improving balance, gait, and physical performance in subjects with mild AD.

Our results are consistent with the published literature about the impact of Wii-Fit exercises on balance and gait in the elderly. In a previous study conducted in an elderly population, improvement in balance (BBS score) and an increase in walking speed were noted with the use of the balance games component of Wii-Fit [18]. In another study conducted on nine elderly patients, an improvement in balance as measured with the BBS was seen along with a decreased risk of falls after five-week use of Wii-Fit [19]. In a study conducted by Williams et al. on community-dwelling fallers over 70 years, improvement in balance (BBS score) was noted in the Wii-Fit group while no improvement in balance scores was noted in the standard care group [20]. In a study conducted in long-term care, residents reported positive experiences playing Wii and rated Wii bowling as a favorite recreational activity [21]. In a small study of 7 patients with AD, Yamaguchi et al. found that use of Video games was beneficial on cognitive and behavioral scales [22].

Our study is different from the above-mentioned studies in that we had a robust arm of a monitored walking program. A typical drawback to walking interventions is the lack of compliance with the recommendation. We overcame that by having the participants walk as a group with research personnel. This ensured that they walked regularly. On average, the walking group had greater exercise compliance (i.e., walked longer than the Wii-Fit intervention time) although this difference in time did not reach statistical significance. This could explain why the Wii-Fit intervention in our study failed to separate from the walking arm. Nonetheless, subjects in the Wii-Fit arm improved as well as the walking arm. People may adhere more to the Wii-Fit intervention since it has been engineered to be socially engaging and entertaining. Wii-Fit is enjoyable, easily accessible, and is not limited by a safe place to walk. One technical difficulty was encountered while using the Wii-Fit that might have reduced the effectiveness of the intervention. In the version of the Wii-Fit used in this study, the actual time spent on exercising was less than the half hour allotted because switching between the exercises consumed time. On an average, only about 22 of the 30 minutes were spent exercising. An upgrade in the newer software, Wii-Fit Plus [23], helps subjects customize and personalize a workout to suit their own needs. They can create their own routine exercises and save them in “my routine exercise” folder which can be accessed in sequence, thus saving time. Future studies will need to determine if this new system improves outcomes.

Unlike the aforementioned studies, we utilized multiple components of the Wii-Fit program such as yoga, strengthening, and balance games in the current study. This approach is supported by findings from a recent Cochrane review [5]. It found that exercise interventions resulted in statistically significantly greater improvements in balance compared to usual activity. Furthermore, interventions involving multiple domains such as gait, balance, coordination and functional exercises, muscle strengthening, and multiple exercise types appeared to have the greatest impact on indirect measures of balance [5]. In another study, gait exercises resulted in improved dynamic balance and gait function [6]. Since our primary outcome measure was that of balance (BBS), a better outcome may be obtained if we specifically targeted balance exercises.

The major strength of the study is the study design, a randomized trial comparing the widely available video game to walking, the most commonly used exercise strategy in the elderly. Limitations of the study include, but are not limited to, the small sample size and inclusion of the BBS as the primary outcome measure which is prone to a ceiling effect, particularly in subjects with higher levels of balance. Other limitations include nonblinded assessors, lack of a usual care control group, and failure to strictly control the intensity of the exercises in each group. The study intervention was also highly monitored and facilitated by the research staff. Having the groups led by peers or having the subjects exercise independently might make the results more translatable to clinical practice.

Future studies could control for the intensity of the exercise by measuring perceived level of exertion, total number of exercises conducted or distance traveled per session, or other parameters. Inclusion of more sophisticated methods for testing balance and gait such as biomechanical assessment might help avoid the ceiling effect of the BBS [24]. The newer version of the Wii-Fit, the Wii-Fit Plus, may be a better tool given its wider variety of exercises and the ability to program the amount of time spent in exercising. Using Wii-Fit in a group setting might have social benefits, which could result in greater improvement in quality of life measures.

## 5. Conclusion

This pilot study demonstrates the safety and efficacy of using Wii-Fit in an assisted living facility in subjects with mild AD. Use of Wii-Fit resulted in significant improvements in balance and gait, and these benefits were comparable to those experienced by the subjects who participated in the robust monitored walking program. These results need to be confirmed in a larger, methodologically sound study. Such a study should probably employ peer-led, family-led, or independently conducted exercise interventions, more sophisticated methods of testing for gait and balance and valid techniques to control the intensity of the interventions.

## Figures and Tables

**Table 1:** Study subject characteristics for the Wii-Fit and walking groups*.

	Wii-Fit group (*N* = 11)	Walking group (*N* = 11)
Age in years, mean (SD)	79.3 (9.8)	81.6 (5.2)
Gender (male/female)	3/8	3/8
Race (Caucasian)	100%	100%
Body Mass Index kg/m^2^, mean (SD)	24.5 (3.5)	26.4 (4.6)
Years of education, mean (SD)	13.8 (2.1)	14.0 (2.0)
No. of comorbidities, mean (SD)	3.2 (1.0)	3.2 (0.9)
Use of assistive device, no.	3/8	4/8
Exercise Time in hrs, mean (SD)	11.1 (3.5)	13.1 (4.3)
Study related adverse events	None	None

*Between group comparisons nonsignificant for all variables (*P* > 0.15).

**Table 2:** The effects of a Wii-Fit and walking interventions on measured outcomes.

Outcome measure*	Group	Baseline mean (SD)	Week 4 mean (SD)	Week 8 mean (SD)	Intragroup change *P* value^†^	Group X time^‡^ Interaction
						*P* value
BBS	Wii-Fit	43.4 (8.9)	47.5 (5.9)	49.6 (5.7)	0.003	0.56
	Walking	41.3 (7.6)	46.9 (6.3)	46.6 (8.7)	0.06	
TT	Wii-Fit	23.5 (3.7)	24.6 (3.4)	25.3 (2.8)	0.013	0.97
	Walking	22.9 (2.6)	24.3 (3.7)	24.9 (3.4)	0.006	
TUG	Wii-fit	14.7 (7.2)	14.3 (6.8)	13.9 (7.9)	0.31	0.52
	Walking	14.9 (4.7)	13.8 (4.2)	12.8 (3.2)	0.07	
ADL	Wii-Fit	22.3 (1.6)	22.5 (1.3)	22.6 (1.3)	0.55	0.11
	Walking	22.0 (2.7)	21.7 (2.5)	21.4 (2.5)	0.11	
IADL	Wii-Fit	11.3 (4.3)	10.5 (2.7)	10.4 (2.8)	0.36	0.11
	Walking	10.9 (3.5)	12.5 (4.4)	11.6 (4.2)	0.53	
QOL-AD	Wii-Fit	36.5 (3.3)	36.3 (3.3)	35.9 (2.8)	0.59	0.61
	Walking	37.3 (4.9)	37.5 (6.1)	35.6 (5.6)	0.03	
MMSE	Wii-Fit	22.6 (4.3)	22.0 (4.1)	22.4 (2.8)	0.93	0.70
	Walking	24.9 (3.6)	25.4 (4.2)	25.5 (4.1)	0.22	

*Abbreviations: BBS: Berg Balance Scale; TT: Tinetti Test; TUG: Timed Up and Go; ADL: activities of daily living; IADL: instrumental activities of daily living; QOL-AD: quality of life-Alzheimer's disease; MMSE: Mini Mental State Examination.

^†^Intragroup change between baseline and week 8, assessed post hoc using the one group paired *t*-test.

^‡^Group by time interaction assessed with RM-ANOVA.
